# Shared decision making and patient-centeredness for patients with poorly controlled type 2 diabetes mellitus in primary care—results of the cluster-randomised controlled DEBATE trial

**DOI:** 10.1186/s12875-021-01436-6

**Published:** 2021-05-15

**Authors:** Anja Wollny, Christin Löffler, Eva Drewelow, Attila Altiner, Christian Helbig, Anne Daubmann, Karl Wegscheider, Susanne Löscher, Michael Pentzek, Stefan Wilm, Gregor Feldmeier, Sara Santos

**Affiliations:** 1 Institute of General Practice, Rostock University Medical Centre, Doberaner Str. 142, 18057 Rostock, Germany; 2 Institute of Medical Biometry and Epidemiology, Medical Centre Hamburg-Eppendorf, Martinistraße 52, 20246 Hamburg, Germany; 3 Institute of General Practice (Ifam), Medical Faculty, Centre for Health & Society (Chs), Heinrich-Heine University Düsseldorf, Moorenstr. 5, 40225 Düsseldorf, Germany

**Keywords:** Diabetes mellitus type 2, Physician–patient relations, Decision making, Health communication, Health services research

## Abstract

**Background:**

We investigate whether an educational intervention of GPs increases patient-centeredness and perceived shared decision making in the treatment of patients with poorly controlled type 2 diabetes mellitus?

**Methods:**

We performed a cluster-randomized controlled trial in German primary care. Patients with type 2 diabetes mellitus defined as HbA1c levels ≥ 8.0% (64 mmol/mol) at the time of recruitment (*n* = 833) from general practitioners (*n* = 108) were included. Outcome measures included subjective shared decision making (SDM-Q-9; scale from 0 to 45 (high)) and patient-centeredness (PACIC-D; scale from 1 to 5 (high)) as secondary outcomes. Data collection was performed before intervention (baseline, T0), at 6 months (T1), at 12 months (T2), at 18 months (T3), and at 24 months (T4) after baseline.

**Results:**

Subjective shared decision making decreased in both groups during the course of the study (intervention group: -3.17 between T0 and T4 (95% CI: -4.66, -1.69; *p* < 0.0001) control group: -2.80 (95% CI: -4.30, -1.30; *p* = 0.0003)). There were no significant differences between the two groups (-0.37; 95% CI: -2.20, 1.45; *p* = 0.6847). The intervention's impact on patient-centeredness was minor. Values increased in both groups, but the increase was not statistically significant, nor was the difference between the groups.

**Conclusions:**

The intervention did not increase patient perceived subjective shared decision making and patient-centeredness in the intervention group as compared to the control group. Effects in both groups might be partially attributed to the Hawthorne-effect. Future trials should focus on patient-based intervention elements to investigate effects on shared decision making and patient-centeredness.

**Trial registration:**

The trial was registered on March 10^th^, 2011 at ISRCTN registry under the reference ISRCTN70713571.

## Background

The rising prevalence of age-associated diseases is a major consequence of increasing life expectancy [1, 2]. Globally, diabetes mellitus is among the most important metabolic disorders and has become a major health care burden [3, 4]. Since 1980, lifetime prevalence of diabetes mellitus showed a major increase with about 6.3% of adults being affected in 2017 [3, 5, 6].

Among patients with type 2 diabetes mellitus (T2DM) registered in the German Disease Management Programme (DMP), 10% show poor control [7]. Studies indicate that various aspects influence the success of treatment and can often compromise adherence. Most common factors include psycho-social stressors, the nature of the disease with e.g., the absence of symptoms, the presence of comorbidities, as well as complex therapy plans poorly adjusted to the patient’s individual situation [8–12]. For the majority of patients, diabetes mellitus is managed by general practitioners (GPs) [13]. From their perspective, treating patients with poorly controlled T2DM is rarely successful and often leads to frustration. This is mainly related to differences in the preferred disease management between patients and doctors. A lack of patient-centred communication reinforces this tendency [14–20].

However, patients increasingly wish to be more involved in decision making [21]. Shared decision making was also shown to be beneficial for physicians` satisfaction with consultations: It allows GPs to gain more insight into the concerns, emotions and needs of their patients [22]. Elicitation and appreciation of patients’ views on their disease allows doctors to better understand their patients’ behaviour and enables them to align treatment plans with their patients’ disease concepts in mind [23–25].

The aim of this study was to investigate the effects of an intervention that facilitates patient-centeredness and shared decision making among GPs and their patients with poorly controlled type 2 diabetes mellitus in German primary care. This paper illustrates the secondary outcomes subjective shared decision making and patient-centeredness.

## Methods

### Trial design

The DEBATE trial is a cluster-randomised controlled trial testing the effect of an educational intervention on the management of patients with poorly controlled T2DM. Primary outcome was the level of HbA1c. Secondary outcomes were patient-centeredness and subjective shared decision making. Data was collected before randomization (baseline, T0), at 6 months (T1), at 12 months (T2), at 18 months (T3), and at 24 months (T4) after baseline. At each point we allowed a maximum of two additional months for data collection e.g., in case where patients could not be reached.

The primary outcome measure (level of HbA1c) decreased significantly both in the intervention and control group. The effect, however, was not significantly different between both groups. Results are published elsewhere [26].

### Recruitment, eligiblity criteria and sampling procedure

For recruitment registers of the regional Associations of Statutory Health Insurance Physicians of the German regions Mecklenburg-Western Pomerania and North Rhine-Westphalia were used. Among participating practices of general practice patients affected by T2DM with an HbA1c level of ≥ 8.0% (64 mmol/mol) within three months prior to recruitment were contacted and included. Patients with one of the following characteristics were excluded from participation: living with a severe comorbidity resulting in an assumed life expectancy below 24 months, inability to give informed consent or lack of sufficient German language skills.

Detailed information on the process of recruitment is published elsewhere [27].

### Intervention

Based on qualitative data from German primary care we know that GPs perceive their patients with poorly controlled T2DM as in denial or refusing to follow advice. In this situation, GPs are sometimes inclined to lower expectations for improvement. In some cases, they also became resigned to the situation [16]. To address this pattern we developed an interventional concept that encourages patient-centered communication and shared decision making.

In a first step, GPs specially trained in patient-centered communication visited enrolled GPs. This peer-visit aimed at sensitizing for patients' concepts of disease and their views, attitudes, and behaviors by using patient-centered communication. In a further step, GPs were encouraged to use the electronic decision-aid (https://www.arriba-hausarzt.de/) to increase shared decision making. The decision-aid uses HbA1c levels and associated risk factors to visualize the probability of experiencing macro vascular events [28, 29]. The effect of antidiabetic medication (such as oral or insulin therapy) and lifestyle changes on cardiovascular events are also shown and serve as a starting point for shared decision making.

A total of 54 GPs were randomized into the intervention group. Out of these after baseline data collection 47 GPs were visited by peers (component 1). Seven GPs were not available for the visit. They received written information material and a phone call. In addition to that, enrolled GPs were provided the chance to take part in a workshop on patient-centered communication. A total of 10 GPs took up the offer (component 2). Please find a detailed description of the intervention components in Tables 1 and 2.

**Table 1:** Intervention description of component 1: outreach educational peer visit (according to TIDieR)

1 Short Name	Educational peer visit
2 Goal and rationale	Improvement of doctor-patient communication and interaction between GP and patient, raising GP awareness for patients with poorly controlled diabetes type 2, their individual agenda and concepts of disease and taking it into account in the process of shared decision making, putting more focus on the patient perspective without overstraining both, doctor and patient
3 Materials	Oral input, computer-based decision-aid tool arriba-debate, peer-to-peer-discussion
4 Procedures	Trained GPs visited participating GPs in their practice. During the visitation, specific problems/factors influencing the doctor-patient-communication and the treatment of patients with poorly controlled type 2 diabetes were discussed with the GP (e.g. different ideas of therapy on GPs and patient's sides resulting in ineffective doctor-patient communication, lack of interest, resignation, frustration, anger). In addition, the peer GP introduced the basics of narrative based communication to the GP and gave individual feedback to patient cases the GP had experienced to be difficult. Additionally, during the visitation, the computer-based decision-aid tool arriba-debate was introduced to the GP. The tool offers patient-targeted visualizations of the effect of possible behaviour changes (e.g. smoking stop, exercise) and therapy (medication) on the individual risk of coronary heart disease under consideration of individual parameters (e.g. sex, age, blood pressure, cholesterol, blood glucose level)
5 Providers of intervention	Trained general practitioners (peers)
6 Mode of delivery	On site visit, oral presentation, introduction of the decision-aid tool and discussion
7 Location	GP practice
8 Frequency	Once following completion of baseline data collection between the 3rd quarter of 2012 and the 1st quarter of 2013; duration approximately 1–1.5 h, total of 47 intervention practices received a peer visit
9 Planned tailoring	No
10 Fidelity enhancement	Memo written by peer

**Table 2:** Intervention description of component 2: additional training on patient communication skills for GPs—optional (according to TIDieR)

1 Short Name	Additional training for GPs to promote patient-centred communication
2 Goal and rationale	Exploration of individual patient expectations, concepts of disease and barriers in the process of shared decision making in patients with poorly controlled diabetes type 2
3 Materials	Theoretical input on narrative-based communication, group training on practical use of these skills, computer-based decision-aid arriba-debate
4 Procedures	Introduction of theoretical background on narrative-based communication (incl. three-step-conversation). Group training sessions (max. 3–4 participants) under considerations of personal experiences and defaulted roles. The issues of the sequences differed, starting with a low-threshold one (e.g. vacation), followed by the experience of an in-acute disease (e.g. cold), ending with a practical oriented issue (e.g. GP as protagonist in the practice). Roles were changed after every session (narrator, asker, observer) to give all participants the opportunity to slip in each role. Subsequently, feedback about the practical implementation was given and discussion about transferability in daily routine was carried out Finally, the computer-based decision-aid tool arriba-debate and its use in daily routine in the GP-practice was discussed
5 Providers of intervention	The training was performed by qualified scientific researchers of the study sites in Rostock, Düsseldorf and Witten
6 Mode of delivery	Single intervention 10 out of the 54 GPs in the intervention group of DEBATE
7 Location	The training was performed in two of the study sites Total of five trainings with altogether 10 GPs were performed
8 Frequency	Each training lasted about 3 h
9 Planned tailoring	No
10 Fidelity enhancement	

### Control

Patients of the control group received care as usual. In Germany, this usually includes measuring levels of HbA1c and consulting the GP two to four times a year. GPs of the control group did not receive any form of intervention.

### Outcomes

Primary outcome was the change of HbA1c level from T0 to T4, which was statistically significant within each group, but not between groups. Results have been published elsewhere [26].

Secondary endpoints included subjective shared decision making (SDM-Q-9 [30]) and patient-centeredness (PACIC-D [31]). Members of the study team collected data by phone at the different points of measurements. Detailed information on data collection procedures was published elsewhere [26, 27].

Analysis of perceived extent of subjective shared decision making from the perspective of T2DM patients was conducted through SDM-Q-9. The questionnaire is based on a 6-stage Likert-scale (0 = does not apply at all; 5 = fully applies). An overall score resulted from the addition of all nine items, giving a scale from 0 to 45. High values indicate that the extent of shared decision making was perceived as high by patients [30].

Patient-centeredness was measured through the short version of the PACIC-D questionnaire that contains 11 items. However, given the age of patients and ease of use, we decided for one modification. Instead of using the scale of the short version (percentage between 0 and 100, in steps of 10 percent), we adapted the five-stage Likert-scale of the long version PACIC-D questionnaire (1 = almost never, 5 = nearly always). The overall score was calculated as the mean. A high extent of patient-centeredness in the GP setting from the patient’s point of view was reflected in a high value [31–34].

### Sample size

Sample size was determined to ensure statistical significance of primary outcomes and was published elsewhere [26, 27].

### Stopping rules

There were no stopping rules within the DEBATE trial.

### Randomization

Randomization was implemented by the study centres after the baseline data collection. Within each study centre, unrestricted randomization was used to allocate the intervention to GPs (cluster level) and their selected patients. Randomization was performed by statisticians not involved in the trial management.

### Allocation concealment mechanism

At recruitment and baseline data collection enrolled GPs were not aware of group allocation.

### Blinding

Study participants were not explicitly blinded. Nonetheless, neither study nurses performing data collection (questionnaires), nor patients were aware of group allocation.

### Measures against bias

To reduce bias related to multiple testing we undertook several preventive measures. These included a standardized interviewer training for all interviewers. Whenever possible, study nurses interviewed the same participants over all points of measurement. Also, questionnaires were not provided before phone interviews and instruments varied to some extent between different points of measurement. Finally, the time interval between measurements was large (six months) and questions always referred to the current day or to the previous six months (see protocol [27]).

### Statistical methods

The chosen versions of SDM-Q-9 and PACIC-D are commonly used in Germany. Initially, descriptive statistics, e.g., mean and standard deviations or absolute and relative frequencies were determined group-wise for each time of measurement.

A longitudinal mixed model with patients and practice as random effects was calculated to account for potential effects of intervention and covariates on changes in SDM-Q-9 and PACIC-D. Follow-ups were incorporated into the model as repeated measurements without restrictions of the covariance matrix. Baseline measurements were included in the model to reduce variance. For control of potential biases, the variables ‘study centre’ and ‘time of measurement’ were included as fixed effects. Marital status, age at initial diagnosis, number of persons in the household and cardiovascular risk were considered as potential covariates on patient level as well as averaged on practice level, complemented by physician’s age. To select these variables, dependent on the scale level of the respective baseline variable, a model with group and study centre as fixed effects was calculated. All variables with a p-value less than 0.2 were included in the next step. In the final set of covariates, that was selected by likelihood ratio based forward selection, only age at diagnosis on patient level was selected as an additional covariate. The time by group interaction was to be tested initially to include this in the model if the interaction was significant. The coefficient test, comparing the adjusted values between the randomized groups, was performed using the direct maximum likelihood as the statistical estimation procedure, which results in unbiased estimators under the missing-at-random-assumption. The analysis was repeated in the per protocol (PP) population. To assess the association between patient-centeredness and subjective shared decision making, we adapted the above described model. SDM was the outcome and changes to baseline in PACIC-D were an additional covariate. The other aspects of the model remained unchanged. Adjusted means with 95% confidence intervals and p values were reported. The significance level was set at two-sided α-level of 0.05. All analyses were conducted with SAS, version 9.4 (SAS Institute Inc. Cary, North Carolina, USA).

## Results

### Recruitment and participant flow

**Fig. 1:**
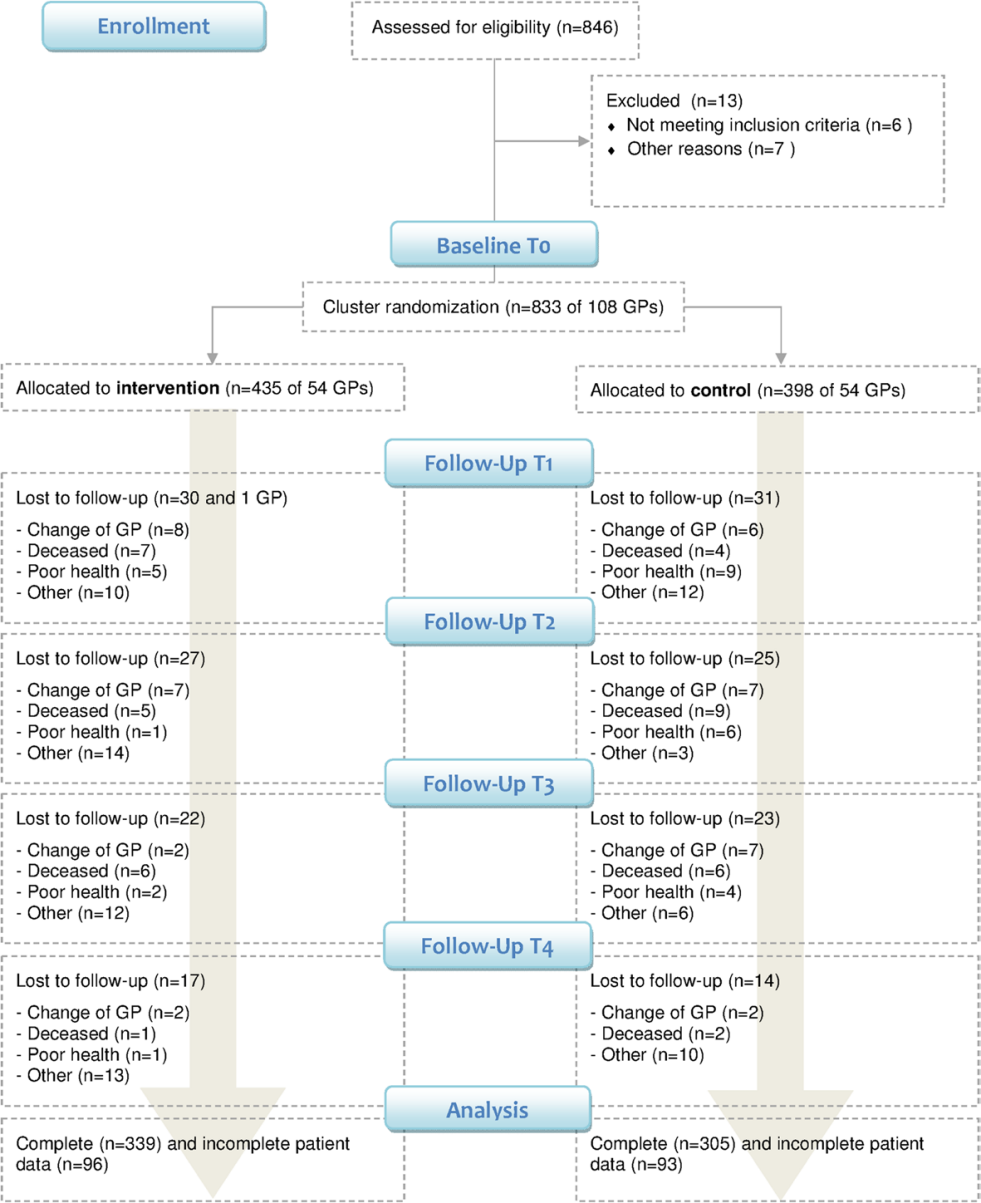
Flow chart of the DEBATE trial

Over the period of 24 months (from 08/2011 to 07/2013), 833 patients of 108 GPs were included in the study. 54 GPs (435 patients) were randomized to the intervention group, and 54 GPs (398 patients) were randomized to the control group. At T4 (24 months), a total of 644 patients remained in the study (339 patients intervention group, 305 control group). Patient drop-outs were 22.1% (intervention) and 23.4% respectively (control). Most drop-outs were related to a change of GP (21.7%), death (21.2%) or a not primarily diabetes related deterioration of the patients' health status (28%). Other reasons included patients' loss of interest in the trial and failed attempts to re-contact trial participants. See Fig. 1 for the detailed flow of participants.

### Baseline data

Basic socio-demographic and health data of participating patients are provided in Table 3. There were no considerable differences between groups.

**Table 3:** Patient baseline data

	**Intervention**		**Control**		**Total**	
	**N**	**%**	**N**	**%**	**N**	**%**
**Number of patients**	435	52.2	398	47.8	833	100.0
**Sex**						
Male	241	55.4	212	53.3	453	54.4
Female	194	44.6	186	46.7	380	45.6
**Age (median)**	65.9		65.8		65.9	
**Marital status**^a^						
Single	46	10.6	41	10.3	87	10.5
Married	273	62.8	229	57.7	502	60.3
Divorced	30	6.9	52	13.1	82	9.9
Widowed	86	19.8	75	18.9	161	19.4
**Living with a partner**^b^						
Yes	291	67.1	252	63.6	543	65.4
No	143	32.9	144	36.4	287	34.6
**Year of diagnosis (mean)**	12.4		10.8		11.6	

^a^ One missing value

^b^ Three missing values

### Outcomes

#### Subjective shared decision making (SDM-Q-9)

**Fig. 2:**
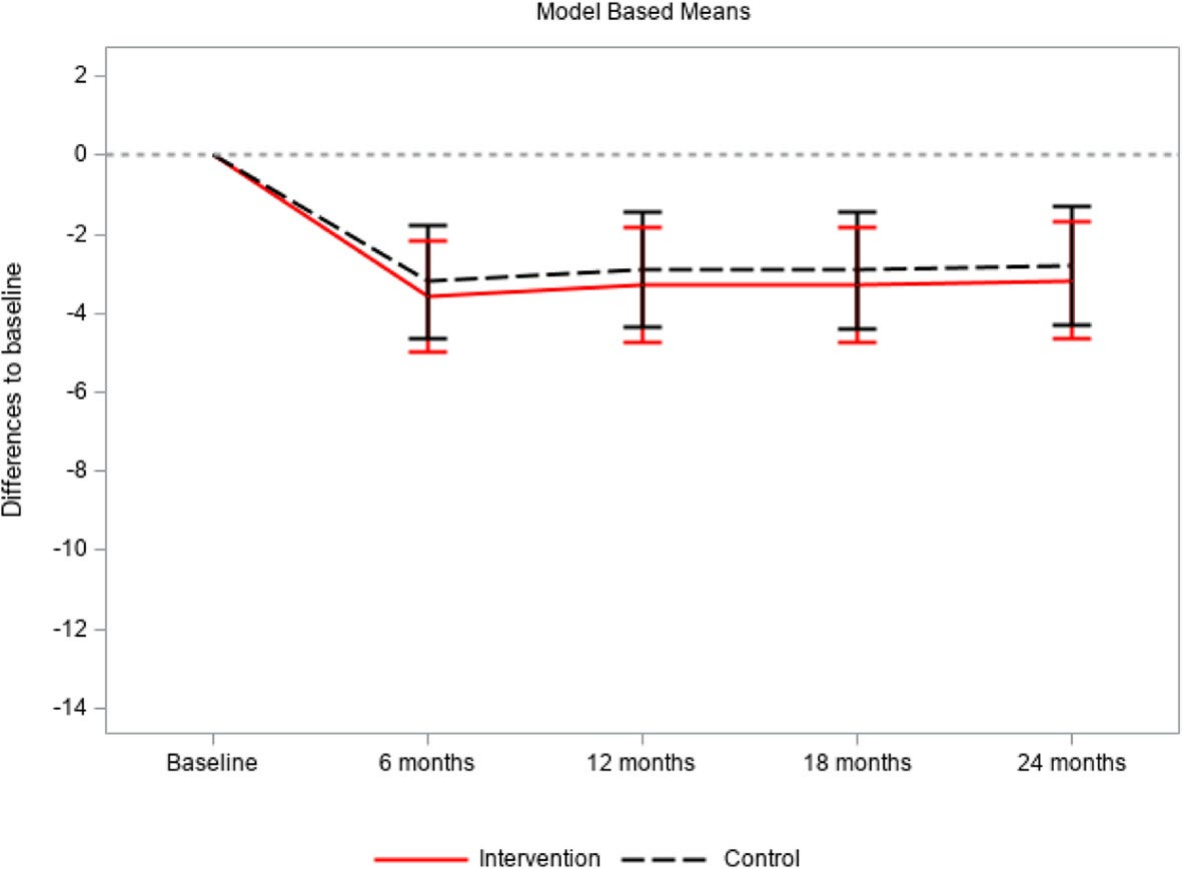
Subjective shared decision making questionnaire (SDM-Q-9): adjusted means in intervention and control group at all times of measurement

The intention-to-treat analysis showed that in the intervention group the mean value of the SDM-Q-9 was 23.68 at baseline. Over the course of the study, the value decreased and then slightly increased while remaining below baseline (20.91 at T4). A similar trend was evident in the adjusted model: the difference to baseline was largest at T1 (-3.57 (95% CI: -4.98; -2.17)) and decreased towards T4 to -3.17 (95% CI: -4.66; -1.69). Within the intervention group, at all times the change in mean SDM-Q-9 values was statistically significant (*p* < 0.0001). Results in the control group were similar: the SDM-Q-9 mean value decreased from 22.42 at baseline to 19.77 at T4. The adjusted difference was maximal at T1 (-3.20 (95% CI: -4.62; -1.78)) and decreased to -2.80 at T4 (95% CI: -4.30; -1.30). Likewise, changes from baseline were statistically significant at all times (*p* < 0.0003). However, between the groups no significant difference was found (*p* = 0.6847). See Table 4 and Fig. 2.

**Table 4:** Shared decision making questionnaire (SDM-Q-9): raw and adjusted means in intervention and control group at all times of measurement, standard deviation, 95% confidence intervals, p-values; model describing the difference between the groups. Intention-to-treat analysis

		Intervention group						Control group							Between group differences			
		Change from baseline						Change from baseline							Intervention group - Control group			
SDM	N	Mean	SD	Adjusted Mean	95% CI		p-Value	N	Mean	SD	Adjusted Mean	95% CI		p-Value	Adjusted Mean	95% CI		p Value
Baseline	395	23.68	13.6					370	22.42	14.4								
6 months follow up	372	19.59	13.4	-3.57	-4.98	-2.17	<.0001	342	19.21	14.5	-3.20	-4.62	-1.78	<.0001	-0.37	-2.20	1.45	0.6847
12 months follow up	338	19.90	14.3	-3.27	-4.71	-1.82	<.0001	312	19.83	14.7	-2.89	-4.35	-1.44	0.0001				
18 months follow up	326	20.83	14.0	-3.28	-4.75	-1.81	<.0001	286	19.64	14.6	-2.91	-4.39	-1.42	0.0002				
24 months follow up	318	20.91	14.4	-3.17	-4.66	-1.69	<.0001	284	19.77	15.2	-2.80	-4.30	-1.30	0.0003

Table 5 shows the results of the per-protocol analysis. Apart from small deviations, the results followed the same trends as in the intention-to-treat analysis.

**Table 5:** Shared decision making questionnaire (SDM-Q-9): raw and adjusted means in intervention and control group at all times of measurement, standard deviation, 95% confidence intervals, p-values; model describing the difference between the groups. Per-protocol analysis

		Intervention group							Control group						Between group differences			
		Change from baseline							Change from baseline						Intervention group - Control group			
SDM	N	Mean	SD	Adjusted Mean	95% CI		p-Value	N	Mean	SD	Adjusted Mean	95% CI		p-Value	Adjusted Mean	95% CI		p Value
Baseline	349	23.36	13.3					370	22.42	14.4								
6 months follow up	328	19.83	13.4	-2.98	-4.44	-1.51	0.0001	342	19.21	14.5	-3.11	-4.50	-1.71	<.0001	0.13	-1.71	1.97	0.8893
12 months follow up	296	20.32	14.2	-2.57	-4.08	-1.07	0.0010	312	19.83	14.7	-2.70	-4.14	-1.27	0.0003				
18 months follow up	289	20.90	13.8	-2.72	-4.24	-1.20	0.0006	286	19.64	14.6	-2.85	-4.31	-1.38	0.0002				
24 months follow up	282	21.05	14.4	-2.57	-4.12	-1.02	0.0013	284	19.77	15.2	-2.70	-4.19	-1.21	0.0004				

#### Patient-centeredness (PACIC-D)

**Fig. 3:**
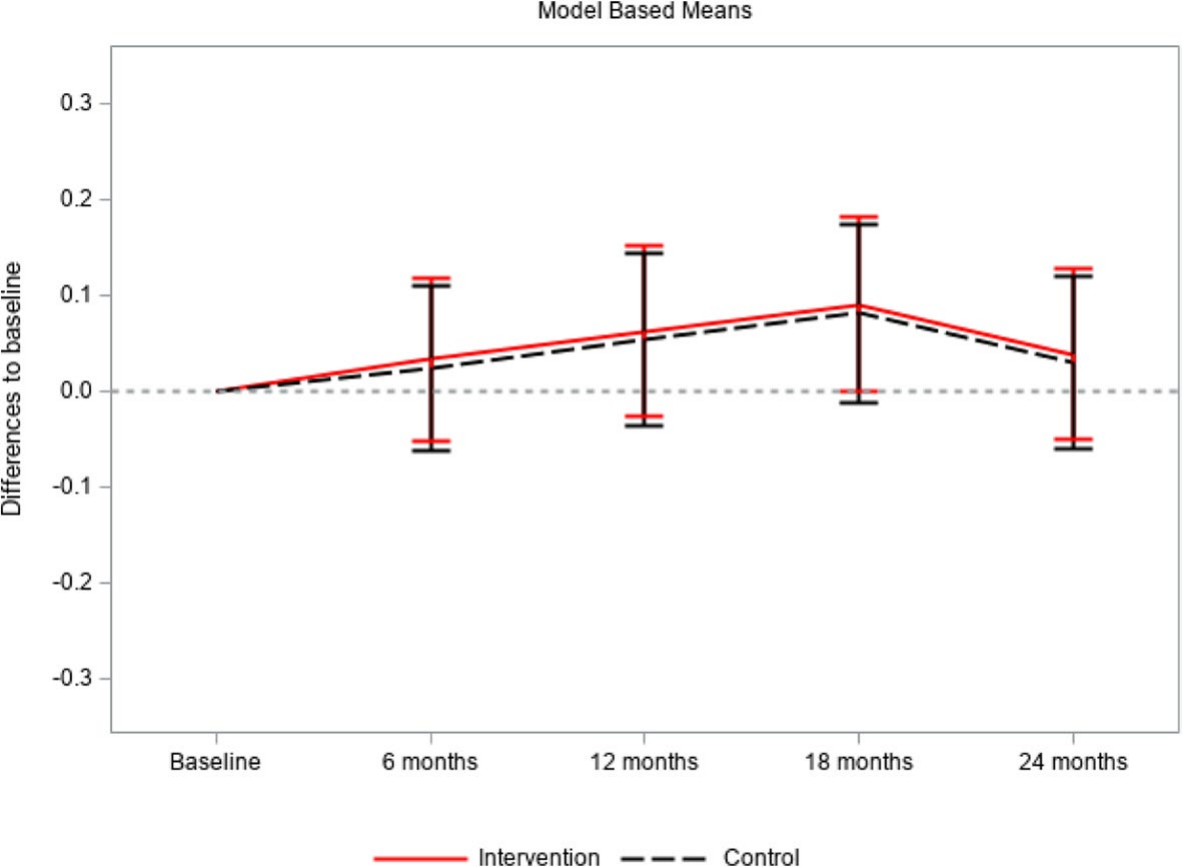
PACIC-D questionnaire: adjusted means in intervention and control group at all times of measurement

As to patient-centeredness, in the intervention group the intention-to-treat analysis showed an average non-adjusted PACIC-D score of 2.42. Over the course of the study, the score slightly increased to 2.52 at T4. The adjusted difference to baseline varied from 0.03 at T1 (95% CI: -0.05; 0.12) to 0.09 at T3 (95% CI: 0.00; 0.18), and finally to 0.04 at T4 (95% CI: -0.05; 0.13). None of the differences were statistically significant. The same was observed in the control group. The non-adjusted PACIC-D score at T0 was 2.39 and reached the highest value of 2.52 at T3. The adjusted difference to baseline varied from 0.02 at T1 (95% CI: -0.06; 0.11) to 0.08 at T3 (95% CI: -0.01; 0.17). None of the corresponding p-values indicated statistical significance. Also, there was no significant difference between the intervention and control group (*p* = 0.8677). See Table 6 and Fig. 3.

**Table 6:** PACIC- D questionnaire: raw and adjusted means in intervention and control group at all times of measurement, standard deviation, 95% confidence intervals, p-values; model describing the difference between the groups. Intention-to-treat analysis

		Intervention group							Control group						Between group differences			
		Change from baseline							Change from baseline						Intervention group - Control group			
PACIC-D	N	Mean	SD	Adjusted Mean	95% CI		p Value	N	Mean	SD	Adjusted Mean	95% CI		p Value	Adjusted Mean	95% CI		p Value
Baseline	404	2.42	0.8					373	2.39	0.9								
6 months follow up	370	2.48	0.9	0.03	-0.05	0.12	0.4340	339	2.41	0.9	0.02	-0.06	0.11	0.5782	0.01	-0.10	0.12	0.8677
12 months follow up	342	2.50	0.9	0.06	-0.03	0.15	0.1627	314	2.47	1.0	0.05	-0.04	0.14	0.2390				
18 months follow up	321	2.53	0.9	0.09	0.00	0.18	0.0518	282	2.52	1.0	0.08	-0.01	0.17	0.0844				
24 months follow up	318	2.52	0.9	0.04	-0.05	0.13	0.3877	282	2.45	1.0	0.03	-0.06	0.12	0.5173

The per-protocol analysis supported the trend observed in the intention-to-treat analysis. There was however one exception: at T3, the adjusted difference of the PACIC-D score to baseline was statistically significant in the intervention group: the PACIC-D score increased by 0.12 (95% CI: 0.02; 0.22, *p* = 0.02). See Table 7.

**Table 7:** PACIC- D questionnaire: raw and adjusted means in intervention and control group at all times of measurement, standard deviation, 95% confidence intervals, p-values; model describing the difference between the groups. Per-protocol analysis

		Intervention group							Control group						Between group differences			
		Change from baseline							Change from baseline						Intervention group - Control group			
PACIC-D	N	Mean	SD	Adjusted Mean	95% CI		p Value	N	Mean	SD	Adjusted Mean	95% CI		p Value	Adjusted Mean	95% CI		p Value
Baseline	355	2.41	0.8					373	2.39	0.9								
6 months follow up	326	2.50	0.9	0.05	-0.04	0.14	0.2484	339	2.41	0.9	0.03	-0.06	0.11	0.5369	0.03	-0.09	0.14	0.6582
12 months follow up	302	2.53	0.9	0.09	0.00	0.18	0.0630	314	2.47	1.0	0.06	-0.03	0.15	0.1649				
18 months follow up	284	2.56	0.9	0.12	0.02	0.22	0.0166	282	2.52	1.0	0.09	-0.00	0.19	0.0500				
24 months follow up	283	2.52	0.9	0.06	-0.04	0.15	0.2349	282	2.45	1.0	0.03	-0.06	0.12	0.4982				

As far as the five dimensions of PACIC-D are concerned, we performed descriptive analyses and found differences between intervention and control group for the dimension "coordination and follow-up". Within the intervention group the dimension-specific mean value increased from 1.95 at T0 to 2.11 at T4. In the control group we observed a decrease from 1.93 at T0 to 1.83 at T1 followed by an increase at T3 (2.02) and a new decline at T4 (1.97). The analyses of the single items supported this observation. However, given the exploratory character of this analysis results need to be treated cautiously.

#### Association of patient-centeredness (PACIC-D) and subjective shared decision making (SDM-Q-9)

Finally, a post-hoc analysis was conducted on whether change in patient-centeredness influenced extent of subjective shared decision making. The model estimated the effect of one unit increase of PACIC-D on subjective shared decision making. The model was adjusted for respective group, study centre, the SDM-Q-9 baseline value, age of initial diagnosis and time of measurement. The model showed that one unit increase of PACIC-D led to an increase of 6.52 units at the SDM-Q-9 scale (95% CI: 5.94; 7.11; *p* < 0.0001).

#### Harms

We did not systematically assess outcomes such as the interaction of medications, cardiovascular events, or death of patients. However, since we tested routine care against routine care with improved GP communication, we did not expect any harms.

## Discussion

### Summary of findings

The DEBATE trial showed that subjective patient reported shared decision making, measured by using the SDM-Q-9 questionnaire, decreased in both intervention and control group between baseline and T4. Although the difference was statistically significant in each group, it was not between the groups. As to patient-centeredness, results were less distinct: Between baseline and T4 the score of the PACIC-D questionnaire slightly increased in both groups, however without being significantly different from baseline. Also, there was no statistically difference between intervention and control group. The analysis of the possible impact of patient-centeredness (PACIC-D) on subjective shared decision making (SDM-Q-9) revealed statistically significance between the two. Thus, a one-point increase of the PACIC-D compared to baseline led to an increase of 6.52 points in the SDM-Q-9 score. Comparison of the PACIC-D results with other studies is not possible given the modification we applied to the instrument. Also, evidence on a clinically meaningful change using both, the SDM-Q-9 and the PACIC-D is limited as scores differ depending on the versions used as well as on cultural context. For these reasons, in general, comparisons are not recommended [35, 36].

### Interpretation in the context of existing literature

The results show that the educational intervention targeting GPs in primary care setting did not lead to a relevant increase in patient reported patient-centeredness and subjective shared decision making. Over the course of the study patients rather felt less involved in shared decision making.

A recent systematic review published in 2018 included 87 studies to determine the effectiveness of interventions on increasing the use of shared decision making by healthcare professionals (HCP). The review showed uncertain evidence. Fifteen studies included specifically evaluated the impact of interventions targeting HCPs—e.g., educational meetings, educational outreach visits, educational material and reminders. The certainty of evidence of these interventions was also low – regardless whether outcomes were measured by observation or reporting by patients [37]. Although there is consensus on the importance and necessity of shared decision making, is seems difficult to measure. The same seems to be true for patient centeredness.

Several studies with a similar outlay as the DEBATE trial showed concurring findings: Within a cRCT Boyd et al. measured the effect of care provided by specially trained nurses (e. g. performing assessments, monitoring care plans, coordinating care givers, and supporting family) on patients’ perceived quality of health. Findings show that the mean PACIC increased over time in both groups, however the overall PACIC was higher for the intervention group. Differences between the groups were not significant [38]. In an evaluation study Szecsenyi et al. tested the effect of participation in a national disease management program for T2DM on patients' perception of chronic illness care. The study found that the difference between intervention and control group was statistically significant for the overall PACIC-score, whereby data was collected only at one time of measurement [39]. Chmiel et al. investigated the effect of a complex educational intervention targeting at practice nurses. The intervention consisted of a comprehensive educational training on medical knowledge about diabetes treatment and general communication skills. In this nested cross-sectional study, among others one outcome measure was perception of diabetes care. The authors proved significant differences between intervention and control group with a higher overall PACIC score for patients of the intervention group over the course of the study compared to those of the control group [40].

Bearing these differing findings in mind, it seems likely that the altered consciousness for patient involvement as part of the social change within the last decades requires broader instruments and concepts to measure shared decision making [37, 41, 42].

### Strengths and limitations

The DEBATE trial benefits from its long time of follow-up: Comparable studies often show a follow-up time considerably lower than 24 months [43, 44]. Also, patients with poorly controlled T2DM, a group that is generally hard to reach, were successfully included in the trial. Number of dropouts was stable in both groups (ca. 20%) and reflected the original sample size calculation. Performing the intervention under real-life conditions in the setting of health care research is another strength of the study.

There are some limiting factors such as measuring subjective perception of shared decision making rather than objective (e.g., through videos of consultations) or the likely inclusion of the most receptive and motivated GPs and patients into the trial. However, in light of the effects shown above we wonder which mechanisms might have led to decreasing levels of perceived shared decision making. With inclusion into the study GPs might have been more motivated to make another attempt with their patients with poorly controlled T2DM. As a result, physicians might have treated their patients more paternalistic and might have put less emphasis on shared decision making. Improvements of HbA1c levels among both groups support this assumption [26]. Moreover, it is possible that peers visiting participating GPs were not able to initiate a change management towards shared decision making. Also, it is likely that the five measurements of shared decision making and patient-centredness over the whole study period increased patient attention towards both aspects (Hawthorne effect). This awareness might have increased patients’ demand for shared decision making and patient-centredness, finally resulting in lower scores. The fact that effects were similar among the intervention and the control group support this assumption.

In retrospect, we recognize in fact discrepancies between the very intensive follow-up on the one side and the comparably surface delivery of the intervention on the other side. Although the trial might have enhanced overall awareness for poorly controlled T2DM among both, patients and physicians, it seems unlikely that it had sustainable impact on GPs counseling behavior. A different and more continuous way of intervention delivery might have been more appropriate. Unfortunately, as highlighted above, to date there is little evidence on the way shared decision making in chronic health care might be improved (and how this improvement might be measured). Légaré et al. showed that it remains uncertain if interventions targeting patients, HCPs or both increase shared decision making [37]. In their systematic review of shared decision making and patient outcomes Shay and Elston Lafata conclude that it remains unclear what leads a patient to report a decision as shared and thus how to foster shared decision making [45].

### Clinical impact

Results did not show a significant difference in subjective extent of shared decision making and patient-centeredness between intervention and control group. In this respect the intervention scheme did not have an effect. Nonetheless, intensive and individual follow-ups, which exceeded the care offered through Disease Management Programmes by far, seem to be supportive. Besides the documentation of laboratory values and the definition of therapeutic outcomes, in particular the patients’ individual perception of and problems with their disease were of interest. The findings might motivate general practitioners to continuously engage with this challenging group of patients and to remain open-minded for new communication approaches, which can help to achieve a continuation of therapy.

### Future research

Instead of delivering physician-focused interventions, future studies might investigate the effect of interventions addressing and empowering both patients and physicians to foster shared decision making and patient-centeredness in diabetes care. In doing so, studies should increasingly focus on the black box “intervention” in regard to their effectiveness or lack thereof. For example, qualitative measures could determine possible hurdles of GPs and patients in changing behaviour.

## Conclusions

The DEBATE study did not result in improved care for patients with poorly controlled T2DM of the trialled intervention group in comparison to the control group. Nonetheless, an improvement in care for this patient population in the primary care setting is desirable. For this reason alone, GPs should be motivated to further engage in this group of patients.

## Data Availability

The datasets generated and analyzed during the current study are available from the corresponding author on reasonable request.

## Abbreviations

GP: General Practitioner
HCP: Health care professional
ITT: Intention-to-treat
PP: Per Protocol
T2DM: Type 2 Diabetes Mellitus
